# Phthalimidoperoxycaproic Acid (PAP) Versus Peroxides and Impact on Dental Enamel After Whitening Treatment: An In Vitro Study

**DOI:** 10.3390/jfb17020104

**Published:** 2026-02-21

**Authors:** Carmen Llena, Lorena Saenz, James Ghilotti, Sofia Folguera, Maria Melo

**Affiliations:** Department of Stomatology, Universitat de València, 46010 Valencia, Spain; llena@uv.es (C.L.); lorenasaenzrd@gmail.com (L.S.); sofia.folguera@uv.es (S.F.);

**Keywords:** dental bleaching, hydrogen peroxide, carbamide peroxide, phthalimidoperoxcaproic acid

## Abstract

Phthalimidoperoxycaproic acid (PAP) emerges as a promising alternative non-peroxide bleaching agent to hydrogen peroxide (HP), offering similar efficacy with potentially less enamel damage. This in vitro study aimed to evaluate and compare the effects of 37.5% HP, 35% carbamide peroxide (CP), and PAP on dental color, enamel surface microhardness, and morphological integrity. Fifty-seven extracted human maxillary incisors were randomly assigned to three groups (*n* = 18). Thirteen teeth per group were used for color evaluation, four for microhardness, and one for surface morphology analysis. Each group received three whitening sessions (three applications per session) according to the manufacturers’ instructions. Color was assessed before and one week after each session using a spectrophotometer. Lightness increased by 7.19 units (HP), 7.11 (PAP), and 4.43 (CP). ΔWI_D_ was 4.48 (HP), 4.16 (CP), and 8.82 (PAP). All agents produced an “excellent” bleaching effect (ΔE_00_ index); only PAP achieved “excellent” values with the ΔWI_D_ index at the end of the study. PAP produced fewer morphological changes on the enamel surface and less reduction in microhardness compared to the untreated control than the other agents evaluated. PAP emerges as an effective alternative for dental bleaching, offering significant color improvement while ensuring minimal alterations to enamel morphology.

## 1. Introduction

Tooth bleaching is a cosmetic procedure designed to enhance dental esthetics by restoring natural tooth color or achieving lighter shades. In modern dentistry, the demand for this treatment has grown exponentially, as tooth color is a primary factor influencing personal appearance and self-confidence [1].

Dental whitening and bleaching agents are categorized into three primary groups based on their chemical composition and mechanism of action. First, oxidizing agents, primarily hydrogen peroxide (HP) and carbamide peroxide (CP), are the most potent compounds and form the core of professional treatments. Second, abrasive and erosive agents, such as silica (abrasives) or weak acids (erosives), remove extrinsic stains through mechanical action. Thirdly, masking substances, including blue covarine and titanium dioxide, create an immediate but temporary visual effect by altering light reflection on the tooth surface [2].

It is important to distinguish between whitening and bleaching. While “whitening” refers to the removal of surface debris and extrinsic stains (typically via abrasives or masking agents found in over-the-counter products), “bleaching” involves the chemical degradation of intrinsic pigments through oxidation [3]. The efficacy of oxidizing agents lies in the release of reactive oxygen species (ROS). These molecules contain unpaired electrons, making them highly unstable and reactive. To achieve stability, ROS attack the long-chain organic chromophores (pigmented molecules) embedded within the dental structure. Through redox reactions, these agents break the conjugated double bonds of the chromophores into smaller, simpler, and less pigmented molecules. Consequently, these smaller molecules absorb less light and reflect more, resulting in a clinically whiter appearance [4,5,6].

Current evidence supports the efficacy of peroxide-based products across various concentrations and protocols. Recent clinical and in vitro studies suggest that a single high-concentration application can be as effective as multiple applications per session, while potentially offering a superior safety profile for the dental pulp [7,8].

Furthermore, a recent umbrella review concluded that there are no significant differences in long-term color change between high-concentration in-office treatments and low-concentration supervised at-home treatments. Both modalities carry a risk of transient dental sensitivity, a common side effect of the oxidation process. Notably, the use of light activation (LED or laser) has been shown to have no significant influence on long-term whitening outcomes, questioning its necessity in routine clinical practice [3].

**Figure 1:**
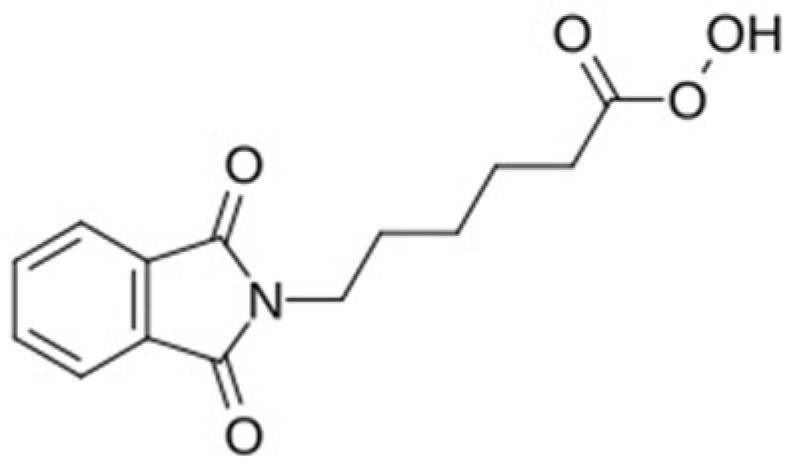
Phthalimidoperoxycaproic acid formula.

Recently, Phthalimidoperoxycaproic acid (PAP) has emerged as a promising bleaching agent, typically formulated as a stable aqueous suspension of PAP crystals. The chemical formula of PAP is C_14_H_15_NO_5_ with a molecular weight of 277.27 g/mol; Figure 1. The key functional groups include the phtalimide (a cyclic imide), the amide-like N-C linkage between the imide nitrogen and the alkyl chain, and the percarboxylic acid (-C(=O)OOH) at the chain terminus, which imparts the molecule’s oxidative character. In peracids such as this, the O–O bond-length in the peroxy group is approximately 1.44 Å, based on computational and spectroscopic data for analogous compounds [9].

Unlike peroxides, the mechanism of action of PAP involves the epoxidation of conjugated double bonds within chromophores. The clinical significance of this pathway is profound. PAP does not generate free radicals during its activation. Extensive literature has linked the release of free radicals from conventional oxidizing agents to adverse effects, including acute dental sensitivity, gingival irritation, and deleterious changes in the enamel’s organic and inorganic matrix [10,11]. By bypassing radical formation, PAP potentially offers a safer profile for the patient.

A critical component in the validation of any bleaching protocol is the assessment of its impact on the enamel structure. Peroxide-based treatments, particularly at concentrations of 35% or higher, have been documented to alter the enamel’s chemical composition and microstructure. To quantify the impact of whitening products on enamel integrity, researchers typically employ a multi-analytical approach: surface microhardness is used to detect the clinical softening of the substrate, a process fundamentally linked to chemical changes in the hydroxyapatite matrix. These changes include alterations in the calcium-to-phosphorus (Ca/P) ratio, which serve as a precise indicator of mineral loss (demineralization) by reflecting the depletion of calcium and phosphorus ions. Furthermore, scanning electron microscopy (SEM) allows for the visualization of resulting morphological alterations, such as increased porosity or erosion [12,13]. Despite these observable changes in vitro, some evidence suggests that the clinical impact may be mitigated by the remineralizing capacity of saliva, which promotes the deposition of minerals back into the porous enamel matrix [14].

Scientific evidence regarding the clinical performance of PAP remains limited. To date, only two in vitro studies have directly compared PAP with 6% HP. These investigations concluded that PAP achieved superior color improvement while causing significantly less structural damage to the enamel [15,16]. At the clinical level, a study using a PAP-based gel (iWhite Instant^®^) demonstrated significant whitening efficacy after a single 20 min application compared to a placebo [17]. However, a significant research gap remains; there are currently no studies comparing the efficacy and safety of PAP against high-concentration peroxides, which are the gold standard in professional in-office treatments.

Given the scarcity of data on PAP’s effects and its comparative performance, this in vitro study aimed to evaluate changes in dental color, enamel microhardness, and surface morphology using 37.5% HP, 35% CP, and PAP-based products. Our working hypothesis was that while all three agents (37.5% HP, 35% CP, and PAP) would produce significant color changes, the PAP-based products would result in the least structural alteration to the enamel, offering a more biocompatible alternative to high-concentration peroxides.

## 2. Materials and Methods

This study was approved by the Human Research Ethics Committee of the “Universitat de València-Spain”. Reference number: 024-ODON-3770202.

### 2.1. Sample Size Calculation

Based on an expected effect size (color change) of 1.2 ΔE_00_ units, a statistical power of 0.8, and a 95% confidence level, a minimum of 13 specimens per group was determined. Five additional specimens were included in each group—four for microhardness analysis and one for morphological evaluation via scanning electron microscopy (SEM).

### 2.2. Study Groups

Fifty-four human incisors extracted for periodontal reasons, free of cracks, structural defects, or carious lesions, were included. Visual inspection of potentially eligible teeth was performed under a clinical microscope Zeiss Kinevo 900S (Zeiss, Oberkochen, Germany) at 40× magnification. In addition, the initial color was determined using the Vita Classic guide (VITA Zahnfabrik GmbH & Co. KG, Bad Säckingen, Germany), with ambient light of 6500 K (daylight). Teeth with A2 or darker were included.

Samples were immersed in 0.5% thymol for one week, cleaned of organic residues, and stored in phosphate-buffered saline (PBS, 0.1%) at a constant temperature of 25 °C until the beginning of the study. No tooth staining was performed.

Canines were not included because the morphology of the crown makes it difficult to take color readings with the spectrophotometer.

Eighteen teeth were randomly assigned to each study group and sectioned 1 mm below the cementoenamel junction. From each group, thirteen teeth were reserved for color change evaluation. The remaining five teeth were sectioned buccolingually through the center of the crown to obtain two halves (mesial and distal) per tooth. To ensure reproducibility and minimize structural damage, each tooth was stabilized in a custom-made acrylic resin jig and sectioned using a water-cooled, low-speed diamond saw (Isomet, Büehler AG, Uzwil, Switzerland) with a 0.3 mm thick disk. This instrumentation allowed precise thickness control and a standardized cutting direction. One tooth (two halves) was randomly designated for SEM analysis, while the other four teeth (eight halves) were assigned for microhardness testing. All specimens were stored in individual PBS-filled vials labeled with an alphanumeric code. The bleaching products used are detailed in Table 1.

### 2.3. Bleaching Protocol

**Figure 2:**
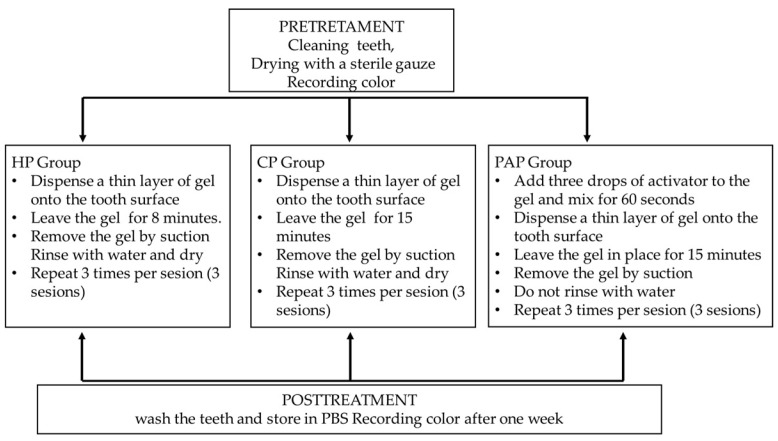
Bleaching protocol: HP: hydrogen peroxide; CP: carbamide peroxide; PAP: phthalimidoperoxycaproic acid.

Before each whitening session, all teeth were cleaned using a prophylaxis paste and brush (Detratrine^®^, Septodont, Lliçà de Vall, Barcelona, Spain). Between the different stages of the study, samples were stored in PBS. Each group underwent three whitening sessions, with three applications of the bleaching agent per session, following the manufacturers’ recommended protocols as outlined in Figure 2. One half of the five samples per group used for the morphological and microhardness study was subjected to the same treatment previously described, while the other half was kept in PBS and served as a control.

### 2.4. Color Change Assessment

**Figure 3:**
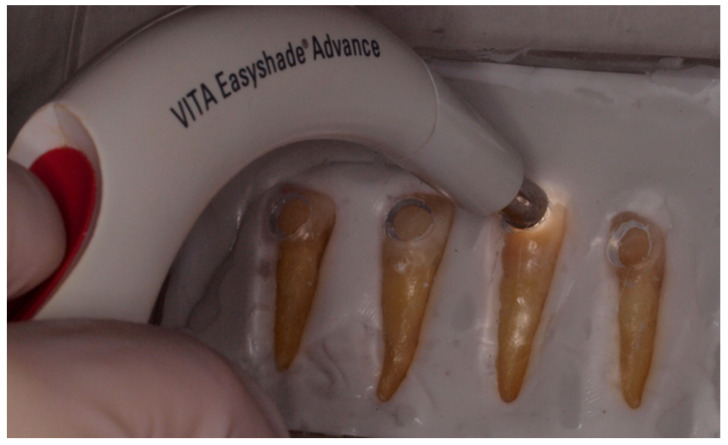
Custom positioning splint to ensure reproducible measurements during the dental shade measurement.

Color measurements were performed using a spectrophotometer (Vita Easyshade, VITA Zahnfabrik, Bad Säckingen, Germany) under standardized ambient lighting. An individualized positioning splint was used for the tip of the spectrophotometer to ensure the repeatability of the measurements. Figure 3 illustrates the position of the spectrophotometer in the color measurement procedure. Color parameters (L*, a*, and b*) were recorded before the first session and one week after each treatment session.

To provide a comprehensive evaluation of the bleaching efficacy, three distinct color indices were calculated: ΔE_ab_, ΔE_00_, and ΔWI_D_. ΔE_ab_ was included to allow comparisons with historical literature and represents the total color distance in the 3D space. However, as this metric lacks perceptual uniformity, the CIEDE2000 formula (ΔE_00_) was also employed; this metric provides a better correlation with the human eye’s perception of small color differences. Finally, the Whiteness Index for Dentistry (ΔWI_D_) was selected specifically to quantify the bleaching effect, as it prioritizes high L* values and low b* values (yellowness), which are critical in dental esthetics.

The detailed formulas are summarized in Table 2. The clinical thresholds for effectiveness according to the literature are described below.

The reference values of the ΔE_00_ index are: ≤0.8 not effective; >0.8 and ≤1.8 moderately effective; >1.8 and ≤3.6 good effectiveness; >3.6 and ≤5.4 very good effectiveness; >5.4 excellent effectiveness [18].

ΔWI_D_ is the difference between two WI_D_ values at different points in the study. The 50:50 perceptibility threshold (WPT) and acceptability threshold (WAT) were established at 0.72 and 2.62, respectively. The reference values of ΔWI_D_ are: ≤2.62 moderately effective; >2.62 and ≤5.2 effective; >5.2 and ≤7.8 very effective; >7.8 excellent [19].

### 2.5. Evaluation of Changes in Vickers Microhardness

As previously described, four samples from each experimental group were allocated for microhardness analysis. Microhardness measurements were carried out on both the treated and untreated halves of each sample using a Struers microhardness tester (Ballerup, Denmark) equipped with a Vickers indenter, applying a load of 300 *g* for 10 s. For each half of every sample, three indentations were performed at predefined and standardized locations, maintaining an adequate distance between indentations to prevent overlapping or interaction effects. The same measurement protocol was applied to all samples. The arithmetic mean of the three indentation values was calculated and used as the representative microhardness value for each sample, ensuring the internal validity and repeatability of the measurements [20]. Before the procedure, the samples were washed with distilled water and dried with blotting paper.

### 2.6. Evaluation of Morphological Changes by SEM

To assess for morphological changes in the enamel, the samples from each group reserved for the morphological study were used. Both the untreated (control) and treated (experimental) halves underwent a rigorous dehydration protocol through a graded series of ethanol concentrations (50%, 60%, 70%, 80%, and 90%, each for 10 min, followed by 100% ethanol for 60 min). To neutralize the ethanol, the samples were immersed in acetone for 30 min and then in amyl acetate for another 30 min for complete dehydration [21]. Following chemical dehydration, the specimens were stored under dry conditions at room temperature.

For conductive enhancement, they were sputter-coated with a gold–palladium alloy using a Polaron Sputter Coater SC 7640 (Leica Microsystems, Wetzlar, Germany). Surface characterization was performed using a SCIOS 2 FIB SEM (Thermo Fisher Scientific, Waltham, MA, USA). Micrographs were captured at magnifications of 3000×, 5000×, 7000×, and 10,000×.

To ensure objectivity, the evaluation was performed by two independent examiners blinded to the experimental groups who described the predominant morphological features based on representative images. To identify treatment-induced topographic changes, symmetrical regions of interest were comparatively analyzed between the experimental and control surfaces.

### 2.7. Statistical Analysis

For the analysis of variables related to color change, normality was assessed using the Shapiro–Wilk test, and equality of variances was checked using Levene’s test. The effect of the treatment group and follow-up time was analyzed using a two-way repeated-measures ANOVA. Equality of covariances and homoscedasticity were verified for each comparison. In all cases, a significance level of *p* < 0.05 was considered. Color indices were compared using ANOVA, taking the baseline condition as a reference and comparing it with the three follow-up time points, with a significance level of *p* < 0.05.

To evaluate for changes in microhardness, a paired *t*-test was used to compare the difference between the microhardness of the two halves of each study sample (treated/untreated), considering a significance level of *p* < 0.05.

## 3. Results

### 3.1. Color Analysis

A multivariate repeated-measures ANOVA was performed to evaluate the effects of follow-up time, treatment group, and their interaction on color coordinates (L*, a*, and b*). Box’s M test confirmed the homogeneity of covariance matrices for all color parameters, indicating that the assumptions for multivariate analysis were met.

For lightness (L*), the effect of treatment time was statistically significant (F = 15.26, *p* < 0.001, power = 0.96) with a large effect size (partial η^2^ = 0.14), indicating an increase in lightness over time regardless of treatment. The effect of the treatment group approached statistical significance (F = 2.92, *p* = 0.06, power = 0.83) with a small-to-moderate effect size (partial η^2^ = 0.07). The interaction between time and group was not significant (F = 0.89, *p* = 0.91, power = 0.06). Bonferroni-adjusted post hoc comparisons showed a significant increase in L* values in the PAP group from baseline to week 1 (mean difference = −6.77; 95% CI: −1.04 to 12.51), week 2 (mean difference = −8.72; 95% CI: −16.52 to −1.19), and week 3 (mean difference = −5.10; 95% CI: −12.64 to −1.42) (*p* < 0.05 for all comparisons). Additionally, in week 2, the PAP group showed significantly higher L* values compared to the HP group (mean difference = −6.88; 95% CI:−13.07 to −0.69; *p* = 0.02).

Regarding the a* coordinate (red–green axis), no significant effects were observed for treatment time (F = 10.36, *p* = 0.13, power = 0.08), treatment group (F = 0.14, *p* = 0.86, power = 0.07) or their interaction (F = 1.40, *p* = 0.87, power = 0.07), all of which were associated with small effect sizes (partial η^2^ < 0.01). Similarly, for the b* coordinate (yellow–blue axis), neither treatment time (F = 0.084, *p* = 0.92, power = 0.05), treatment group (F = 0.46, *p* = 0.63, power = 0.11) nor their interaction (F = 0.31, *p* = 0.73, power = 0.09) showed statistically significant effects, with consistently small effect sizes.

Overall, changes in color were mainly driven by increases in lightness over time, particularly in the PAP-treated group, while chromatic components (a* and b*) remained stable. The low statistical power observed for some interaction effects suggests that nonsignificant findings should be interpreted with caution.

Using Bonferroni’s test, it was found that in the group treated with PAP, lightness increased significantly from baseline throughout the three weeks of treatment. Additionally, after the second week of treatment, the PAP group showed a significant increase in lightness compared to the HP group. The a* and b* variables did not undergo significant changes either between groups or within each group. The data are shown in Table 3.

**Figure 4:**
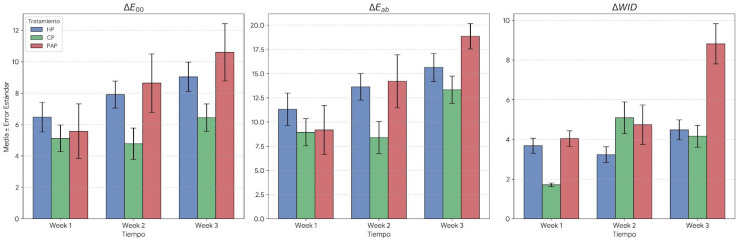
Color indices for each follow-up time based on baseline color. HP: hydrogen peroxide; CP: carbamide peroxide; PAP: phthalimidoperoxycaproic acid.

Color indices ΔE_ab_, ΔE_00_, and ΔWI_D_ were calculated by determining the differences between baseline values and those obtained after each treatment session. As shown in Figure 4, bleaching was found to be between very effective and excellent in all groups at the end of the treatment, based on the ΔE_ab_ and ΔE_00_ indices. Analysis using the ΔWI_D_ index at the end of the follow-up period indicated good effectiveness for HP, very good for CP, and excellent for PAP. Only for the ΔEab index at the end of the treatment period were significant differences found between CP and PAP.

### 3.2. Changes in Vickers Microhardness

A significant reduction in the Vickers microhardness of enamel was observed after treatment with the three bleaching products under study. The effect was most pronounced in HP, showing the greatest absolute difference between untreated and treated samples (413.15 ± 19.62/335.90 ± 23.64; *p* < 0.001). For CP, the values were (302.40 ± 40.84/254.90 ± 40.52; *p* = 0.004), and for PAP (273.60 ± 43.86/226.80 ± 37.85; *p* < 0.001).

### 3.3. Morphological Changes (SEM)

**Figure 5:**
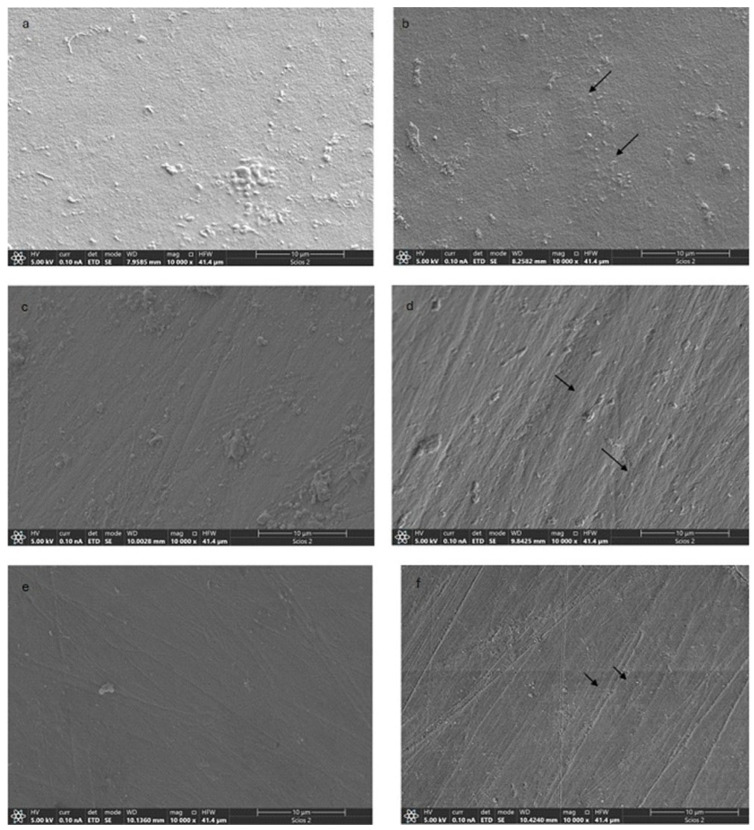
Enamel changes (SEM): Untreated enamel surfaces (**a**,**c**,**e**); enamel treated with 37.5% hydrogen peroxide (**b**); enamel treated with 35% carbamide peroxide (**d**); enamel treated with phthalimidoperoxycaproic acid (**f**).

SEM revealed distinct differences in surface morphology between untreated enamel and enamel treated with the different bleaching agents. Untreated enamel surfaces (Figure 5a,c,e) consistently appeared smooth and homogeneous, exhibiting only faint linear features characteristic of the natural enamel topography and no evident surface defects. In contrast, enamel treated with HP (Figure 5b) showed clear surface alterations, including an increased surface irregularity and the presence of micro-porosities and localized surface defects. Similarly, CP-treated enamel (Figure 5d) exhibited a rougher surface morphology, with more pronounced linear irregularities and localized micro-depressions. Enamel treated with PAP (Figure 5f) also demonstrated morphological changes, characterized by increased surface roughness, accentuated linear grooves, and discrete surface defects (indicated by arrows).

Overall, all bleaching treatments resulted in a less uniform enamel surface compared to the untreated controls, with observable differences in surface texture and microstructural integrity. The most pronounced surface alterations were observed in the HP-treated enamel (Figure 5b), followed by CP (Figure 5d), while PAP-treated enamel (Figure 5f) exhibited comparatively milder morphological changes.

## 4. Discussion

### 4.1. Color Analysis

Tooth bleaching is one of the most frequent esthetic demands in contemporary dentistry, where the comparative evaluation of the efficacy and safety of bleaching agents remains an area of intense research [11]. This study addresses a gap in the dental literature by providing comparative evidence on PAP, an agent whose mechanism of action and efficacy have been less studied than those of peroxides.

The present investigation aimed to test the hypothesis that the use of bleaching products based on high concentrations of HP, CP, and PAP generates significant changes in color, microhardness, and morphology of dental enamel, with PAP causing fewer structural alterations compared to peroxides. This hypothesis was corroborated based on the results of the present study.

Color changes were evaluated using a spectrophotometer, employing the CIELab chromatic coordinates. Lightness was the parameter that showed the most changes in all study groups, obtaining an increase of 7.19 units for the group treated with HP; 7.11 in the group treated with PAP; and 4.43 in the group treated with CP. The maximum value for all color indices was obtained in the group treated with PAP at the end of the treatment period. Statistically significant differences were only found in the ΔE_ab_ values after the third week of treatment between the PAP group and the CP group. “Excellent effectiveness” levels were exceeded in all treatment groups with the ΔE_ab_ and ΔE_00_ indices, and only in the PAP group with the ΔWI_D_ index, remaining at values between 2.6 and 5.2, was considered effective [18,19]. This may indicate that the WI_D_ index is more sensitive in assessing changes in tooth color and that it more comprehensively evaluates the color alteration process and its acceptability.

The most relevant finding was the significant impact of the time factor on lightness (L*). The progressive improvement of L* in the PAP group from baseline through the third week suggests consistent and effective action. Interestingly, while the ANOVA did not show overall significant differences between groups for lightness (*p* = 0.06), the Bonferroni post hoc analysis revealed that the PAP group experienced a significant reduction in lightness compared to the HP group after the second week. This may indicate a distinct optical behavior or saturation point between peroxide and non-peroxide formulas.

In contrast to lightness, the chromatic components a* (red/green axis) and b* (yellow/blue axis) showed no significant changes influenced by time or treatment type. This suggests that the observed bleaching was primarily due to an increase in tooth lightness rather than a drastic alteration in the saturation of specific pigments. The low statistical power observed for these interactions (power\0.07) reinforces that variations in a* and b* were marginal compared to the change in value (lightness).

An in vitro study demonstrated the superiority of PAP over 6% HP, with an increase in SGU units using the Vita Bleaching guide, of 4.82 with 6% HP and 8.13 with PAP after three applications of each product [15]. A clinical study used a gel containing PAP versus a placebo gel, finding an improvement of around 3 SGU units after a single application of the product for 15 min [17]. In this study, with 37.5% HP and 35% CP, we found a ΔWI_D_ difference of 4.48 and 4.16, respectively, while with PAP, it was 8.82. Therefore, the present study corroborates the superiority of PAP in terms of bleaching effect compared to conventional peroxides. Comparing different concentrations of PAP and HP for 7 days, it was shown that a 5% PAP gel provided equivalent ΔE_ab_ results to 3% HP, and 12% PAP concentrations were equivalent to 8% HP. The ΔE_ab_ values were superior, and a greater bleaching effect was found with the 12% concentration [16]. In the present study, the ΔE_ab_ values were lower than those reported by the aforementioned authors, which could be due to differences in the substrate; in our case, human teeth were used, while they used pre-stained bovine teeth. The measurement method was also not the same; they used a colorimeter, while in the present study, a spectrophotometer was used.

HP concentrations between 35–38% and CP between 37–35%, respectively, in clinical studies, found changes analyzed with CIE2000 in ranges similar to those found in the present study [22,23]. The consistency of the studies suggests that, regardless of small variations in the concentration of the bleaching agent, the results with high-concentration products are similar.

### 4.2. Microhardness Analysis

It is important to note that the microhardness findings presented here must be considered preliminary. A formal sample size calculation was not performed specifically for the evaluation of differences in Vickers hardness between the experimental groups. Consequently, this study only reports comparisons within each study group (longitudinal changes) and does not provide statistical comparisons between the three treatments. Furthermore, the analysis was conducted using a limited sample size of only four specimens per group. While these data offer a valuable initial insight, the small “*n*” and the lack of intergroup analysis limit the ability to determine which agent is safest for the enamel relative to the others. These results should therefore be interpreted with caution and serve as a pilot for future research.

The evaluation of Vickers microhardness is a critical parameter in assessing the safety of bleaching agents, as it reflects the potential mineral loss or structural alterations in the dental enamel [20]. In this study, the results indicated that despite the significant whitening effect observed in all groups, the microhardness values did not show drastic fluctuations that would suggest severe demineralization. This stability is particularly relevant for the PAP group, as it suggests that its non-peroxide mechanism effectively lightens the tooth structure without compromising the inorganic matrix of the enamel as significantly as high-concentration peroxides might.

The results of this study align with previous findings, suggesting that high-concentration bleaching gels can depress enamel microhardness. Specifically, a more pronounced reduction was observed with HP compared to CP, a trend also described by Buzatu et al. [24]. Nevertheless, the data obtained in the present study suggest that the specific bleaching protocols—in terms of both application time and concentration—were sufficiently balanced to maintain the structural integrity of the enamel surface across all experimental groups.

Furthermore, other research has demonstrated an increase in microhardness following the application of PAP, contrasted with a reduction when using HP at various concentrations [16]. It is important to note that those authors utilized a non-commercial PAP formulation. This may account for the discrepancies with our results; commercial products, such as the ones used in this study, often include sodium hydroxide to alkalize the formulation. While sodium hydroxide is a potent alkaline agent used to stabilize pH, differences in concentration and the presence of other buffering agents in commercial versus experimental formulas can significantly influence the resulting surface morphology and mineral density of the enamel.

### 4.3. Enamel Morphological Changes

While these findings provide valuable insights, they should be considered preliminary due to the relatively small sample size, which may limit the generalizability of the SEM data.

The SEM analysis demonstrated that all bleaching agents induced detectable alterations in enamel surface morphology when compared to untreated controls. Among the evaluated treatments, HP produced the most pronounced surface changes. These differences may be related to the distinct chemical mechanisms and oxidative potentials of the bleaching agents. HP, due to its higher reactivity, has been associated with greater interaction with the enamel surface, potentially leading to more noticeable morphological alterations [24,25,26,27].

CP decomposes into HP and urea, which may jointly influence enamel surface morphology. The gradual release of HP results in oxidative interactions with the enamel surface, while urea further decomposes into ammonia, leading to an increase in local pH. This alkalinizing effect may partially counteract enamel demineralization, thereby limiting the severity of surface alterations [28]. As a result, CP tends to produce intermediate morphological changes when compared with HP, which is consistent with the SEM observations of the present study.

In contrast, PAP is a peracid that exerts its bleaching effect through a different oxidative mechanism and is reported to be active at near-neutral pH. This may limit mineral dissolution and reduce the extent of surface damage. Accordingly, PAP-treated enamel in the present study exhibited comparatively milder morphological changes, with greater preservation of surface continuity when compared to HP- and CP-treated enamel. A previous study showed minor changes compared to those found in the present study, which can be justified, as mentioned regarding microhardness, by the absence of HP used in that study [16,29].

The results obtained regarding color change, with excellent ΔWI_D_ values after a single session of three PAP applications, together with very slight structural changes in the enamel after three sessions of three applications each, suggest that PAP could be a promising alternative to conventional bleaching agents. These results confirm our working hypothesis.

Its long-term efficacy requires further investigation due to the limited scientific evidence available in the current literature. The scarcity of previous studies directly comparing PAP with other bleaching systems makes it difficult to contextualize these findings. Future research should explore its chromatic stability, side effects, and optimal application protocols. Furthermore, it is necessary to implement clinical studies to establish the behavior of this novel bleaching agent under clinical conditions.

### 4.4. Limitations of the Study and Future Perspectives

Despite the relevance of the findings presented, several limitations of this study must be acknowledged. The in vitro nature of the experimental design does not fully replicate the complex oral environment, where factors such as saliva, temperature fluctuations, dietary habits, and mechanical forces may influence both bleaching efficacy and enamel response. Therefore, extrapolation of these results to clinical conditions should be performed with caution.

The limited sample size, particularly for the microhardness and SEM analyses, restricts the statistical power of the study and precludes robust intergroup comparisons. As previously stated, the microhardness analysis should be regarded as preliminary, serving primarily as a pilot evaluation rather than a definitive assessment of enamel safety among the different bleaching agents.

Specimens were stored in PBS, which does not fully replicate the dynamic oral environment. The absence of salivary components, remineralization potential, and pH fluctuations may have influenced the extent of enamel surface and microhardness changes observed, potentially overestimating these effects compared to clinical conditions. Nevertheless, the use of PBS allowed for a standardized and controlled evaluation of the direct effects of the bleaching agents. Future studies incorporating artificial saliva and/or pH-cycling models are recommended to enhance clinical relevance.

Additionally, this study focused on short-term color changes and immediate enamel alterations. Long-term outcomes, such as color stability over time, cumulative effects of repeated bleaching cycles, and potential delayed enamel changes, were not assessed. These aspects are clinically relevant and warrant further investigation.

Future studies should therefore include larger sample sizes with appropriate power calculations, as well as longitudinal designs to evaluate the durability of whitening effects and the long-term impact on enamel integrity. The inclusion of in situ and clinical trials is essential to confirm the behavior of PAP-based bleaching systems under real oral conditions, including their effects on dental sensitivity, patient comfort, and overall treatment satisfaction. Moreover, future research should explore different PAP concentrations, application protocols, and formulations, as well as their interaction with remineralizing agents, to optimize the balance between esthetic efficacy and biological safety.

## 5. Conclusions

Based on the results of the present study and taking into account the limitations of in vitro studies, it can be concluded that while all evaluated agents were effective, treatment with PAP offered superior whitening efficacy according to the ΔWI_D_ index, achieving an ‘excellent’ rating that surpassed the results of HP and CP at the end of the three-week follow-up. Although the results on microhardness and morphological changes should be considered preliminary, enamel microhardness was reduced with all bleaching products used, with the highest reduction observed in the HP-treated group. All bleaching agents evaluated induced detectable changes in enamel surface morphology. HP produced the most pronounced alterations, CP showed intermediate effects, and PAP resulted in comparatively milder surface changes, suggesting a greater preservation of enamel surface integrity.

## Figures and Tables

**Table 1:** Composition of bleaching products used.

Group	Product/Manufacturer	Composition
HP	Pola Office 37.5%^®^ SDI, Bayswater (Melbourne), Victoria, Australia	37.5% hiydrogen peroxide, additives, glycerol, water and 0.5% flavorings
CP	Pola Day CP 35%^®^ SDI, Bayswater (Melbourne), Victoria, Australia	35% carbamide peroxide, 40% of water, 0.5% of flavoring, 24.5% of thickening agents
PAP	Brilliant Lumina^®^ Coltene, Altstätten, Switzerland	Gel: 12% phthalimidoperoxycaproic acid, glycerin, xanthan gum, monosodium citrate, PVM/MA copolymer, sodium hydroxide, C12–15 pareth-3, activator, water, sodium hydroxide

HP: hydrogen peroxide; CP: carbamide peroxide; PAP: phthalimidoperoxycaproic acid.

**Table 2:** Detailed formulas.

Index Formulas
ΔE_ab_ = [(L_after − L_before)^2^ + (a_after − a_before)^2^ + (b_after − b_before)^2^]^1/2^.
ΔE_00_ = {[ΔL∕(K_L_ S_L_)]^2^ + [ΔC∕(K_C_ S_C_)]^2^ + [Δh∕(K_H_ S_H_)]^2^ + R_T_(ΔC/(K_C_ S_C_) (Δh/(K_H_ S_h_H}^1/2^.
WI_D_ *=* 0.511L − 2.324a − 1.100b.

L: lightness; a: red-green distance; b: yellow-blue distance. K_L_ (lightness weighting factor): Adjusts the influence of light difference. S_C_ (chroma weighting factor): Adjusts the influence of saturation. K_h_ (hue weighting factor): Adjusts the influence of hue. R_T_ (rotation term): Its specific function is to correct the problematic interaction between chroma and hue in the blue area (around 275°). S_L_ (clarity weighting function): Compensates for the human eye’s lack of uniformity in response to changes in light. S_C_ (chroma weighting function): Compensates for perception based on saturation (we are less sensitive to chroma differences when the saturation is very high). S_H_ (hue weighting function): Compensates for perception based on a specific hue. ΔL (lightness difference): The calculated difference in lightness. ΔC (chroma difference): The difference in color intensity. ΔH (hue difference): The difference in hue or “color” itself.

**Table 3:** Distribution of L, a*, and b* values for each follow-up time and study group.

*n* = 13	Group	Mean	SD		Mean	SD		Mean	SD
L0	HP	83.66	6.98	a0	0.43	2.07	b0	23.13	8.28
	CP	88.13	7.05		0.53	1.49		25.49	8.35
	PAP	87.37 ^a.b.c^	9.44		0.50	1.76		26.54	8.02
L1	HP	88.76	5.81	a1	−0.08	2.55	b1	22.20	8.04
	CP	90.16	7.03		−0.21	2.28		24.56	8.76
	PAP	94.15 ^a^	4.36		−0.43	2.8		23.16	9.82
L2	HP	89.21 _1_	5.79	a2	−0.20	1.82	b2	21.12	6.93
	CP	92.10	5.54		−0.70	1.57		23.32	5.76
	PAP	94.10 ^b^_1_	7.36		−0.79	1.68		21.23	7.32
L3	HP	90.85	6.85	a3	−0.63	1.97	b3	21.06	8.03
	CP	92.56	5.08		−0.78	1.46		22.12	6.6
	PAP	94.48 ^c^	4.35		−0.5	0.96		20.03	6.7

HP: hydrogen peroxide; CP: carbamide peroxide; PAP: phthalimidoperoxycaproic acid. The same superscript letter indicates significant differences within the treatment group at different time points. The same subscript number indicates significant differences between treatment groups within each time point.

## Data Availability

Data are contained within the article.
